# Demographic and prosocial intrapersonal characteristics of biobank participants and refusers: the findings of a survey in the Netherlands

**DOI:** 10.1038/s41431-020-0701-1

**Published:** 2020-07-31

**Authors:** Reinder Broekstra, Judith Aris-Meijer, Els Maeckelberghe, Ronald Stolk, Sabine Otten

**Affiliations:** 1 Department of Epidemiology, University Medical Center Groningen, University of Groningen, Groningen, The Netherlands; 2 Wenckebach Institute for Medical Education and Training, University Medical Center Groningen, University of Groningen, Groningen, The Netherlands; 3 Department of Social Psychology, Faculty of Behavioral and Social Sciences, University of Groningen, Groningen, The Netherlands

**Keywords:** Genetics research, Epidemiology

## Abstract

Research in genetics relies heavily on voluntary contributions of personal data. We aimed to acquire insights into the differences between participants and refusers of participation in a Dutch population-based biobank. Accordingly, we assessed the demographic and prosocial intrapersonal characteristics of respondents who participated (*n* = 2615) or refused to participate (*n* = 404) in the Lifelines biobank and databank. Our results indicated that health-related values critically influence participation decisions. The participation threshold for Lifelines was determined by an absence of health-related values and of trust in government. Therefore, considering these factors in communication and recruitment strategies could enhance participation in biomedical research. No indications were found of a stronger general prosociality of participants or their trust in researchers beyond the context of biobanking. This emphasizes the contextual understanding of the decision of participation in biobanking. Our findings may contribute to improving recruitment strategies by incorporating relevant values and/or highlighting prosocial benefits. Moreover, they foreground the need to address trust issues in collaborations between data repositories and commercial companies. Future research should explore how prosocial intrapersonal characteristics drive participation and withdrawal decisions and relate to contextual attributes.

## Introduction

Genetic and biomedical research rely heavily on voluntary contributions of personal data [1]. Simultaneously, an increase in calls for data has led to mounting concerns about data security and privacy [2]. These concerns might lead to lower willingness to participate in biomedical research [3–6]. Therefore, a study of the differences between those who accept and decline invitations to participate in a biobank could yield valuable insights into effective strategies for recruiting participants [7].

Studies have identified various factors that indicate whether people are willing to participate in biobanks or not. There is ample evidence that several demographic characteristics are associated with the intention to participate in biobanks, e.g., being highly educated [8–11], having a partner [6, 12], being non-religious [10, 13] and having better (self-reported) health [8, 14]. Other demographic characteristics are more inconsistently associated, such as being older [3, 8, 15] or younger [10, 11] vs. no age-effect [9, 16, 17]; being male [3, 17] or female [16] vs. no gender effect [9, 10].

Psychological characteristics, such as trust in and concern for others, provide a stable pattern of associations with willingness to participate. Studies report that participation willingness in biobanks is strongly associated with having greater societal trust [3, 8], with higher levels of social engagement [18] and with being registered as organ or blood donor [8]. Societal trust refers to a generalized trust in the government as system or institution [19], and in unknown other citizens [20]. Other strong associations with participation have been found for factors specific to the biobanking domain, such as trust in biobanks [6, 9] or positive attitudes towards biobanking in general [3, 21, 22], and towards biomedical research [21, 22].

Attitudes, values and behaviours reflecting cooperative tendencies and confidence in cooperation—which we here summarize as prosocial intrapersonal characteristics—independently contributed to willingness to participate in biobanking [9, 21]. The key role of these prosocial characteristics is in line with studies emphasizing the importance of prosocial motives for participation in biobanking [3, 4, 23]. Moreover, similar associations were found for prosocial characteristics with decisions about cooperation in other societal contexts, for example, participation in voluntary work [24] or in smart energy systems [25]. Especially, societal trust and domain-specific values, e.g. concern for environment, proofed to be robust predictors in these contexts [26–28].

Furthermore, psychological characteristics seem to explain non-participation, and in particular refusal, better than demographic characteristics. For example, a study in the USA found that women and older patients were more likely to respond to recruitment, but these demographics were not associated with refusal. Instead, people refusing participation in biobanking looked, just as participants, more often and more actively for information about biobanking than non-participants not responding to recruitment request, while participants as well as both non-participant groups had the same prior knowledge [16].

It is possible that thresholds for participation arise due to competing values about society or concerns about biobanking. A recent study in Finland showed that donating blood for biobanking purposes was perceived with more concerns than donating blood for treatment of patients [29]. Trust reduced complexity of decisions about participation by perceiving fewer risks [19, 30]. A lack of trust indicates an increased anxiety about harm [31]. This anxiety may be exacerbated when sharing data with biobanks, in particular involving commercial enterprises or researchers. Studies showed that refusal in biobanking is related to societal and individual concerns, such as fear of discovering possible genetic predisposition to certain diseases leading to social stigma [21]; worry that information may be used against personal interest resulting into discrimination [16, 21, 30]; or concerns about commercial purposes eventually derogating public goods [21, 23, 30].

Together, the evidence summarized above suggests that psychological characteristics could be relevant predictors of both participation and refusal in biobanking. We investigated people who either participated or explicitly refused to participate in a Dutch population-based biobank. We aimed to contribute insights into (non-)participation in biobanking by simultaneously investigating demographic and prosocial intrapersonal characteristics to elucidate their independent roles in the decision to participate or refuse participation, regardless of the motives for these decisions.

## Materials and methods

### Procedure and participants

We conducted an online survey of a sample of the general population in the Northern provinces of the Netherlands (*n* = 3019), comprising 2615 participants in a Dutch biobank, and 404 citizens who refused or signalled refusal to contribute to Lifelines or similar large-scale centralized data repository for scientific medical research. Our sample of participants was recruited via Lifelines in May 2018. The response rate for the invitation of this additional study was 22.2% without incentives. Lifelines recruited its participants between 2006 and 2013 from the general population in the northern provinces of the Netherlands, and providing a representative sample (*n* = 167,000) [12, 32]. Our sample of refusers was recruited among a representative respondents’ panel of the Northern Dutch population via the DirectResearch EUpanel (*n* = 30,600*)* in August of the 2018. These respondents received a small financial incentive for their studies, resulting in a response rate of 41.4%, respectively. Both organizations applied the same recruitment protocol and stratified the samples for sex and age. The subsample of biobank participants was provided by Lifelines themselves, since they exclusively recruit data from their participants. These participants consented between 2006 and 2013 to participate in a longitudinal prospective cohort study of onset, risk factors and course of chronical diseases [32]. Refusers stemmed from the DirectResearch sample from the northern general population. Self-reported refusal was measured via two items at the beginning of our questionnaire. First, these were people who indicated that they did not participate “in large scale medical scientific studies, for example Lifelines” (*n* = 1738). Next, within this broader sample of non-participants, we identified refusers via their affirmation of the statement “I did not want/would not want to participate in this type of research” (*n* = 404). The mean age of Lifelines’ respondents was 56 (SD = 15.88) with 50.5% being male, whereas the mean age of DirectResearch’ respondents after exclusion was 56 (SD = 14.18) with 48.5% being male (Table 1 and Supplementary Table 1).

**Table 1:** Demographic characteristics and self-reported health of participants and refusers of a Dutch population-based biobank.

	Participants		Refusers		*p*
Age	56.27	(15.88)	56.09	(14.18)	= 0.83
Gender					= 0.47
Male	1291	(50.5%)	179	(48.5%)	
Female	1264	(49.5%)	190	(51.5%)	
Marital status					≤ 0.01
Registered partner	2078	(79.5%)	269	(66.6%)	
No registered partner	536	(20.5%)	134	(33.4%)	
Educational level					≤ 0.01
High	1083	(41.4%)	189	(46.8%)	
Moderate	938	(35.9%)	50	(37.1%)	
Low	559	(21.7%)	60	(14.9%)	
Paid Job	1460	(55.8%)	205	(50.7%)	= 0.07
Religious	972	(37.2%)	140	(34.7%)	= 0.48
Residence					= 0.04
Rural	2019	(77.2%)	295	(73.0%)	
Urban	537	(20.5%)	102	(25.2%)	
Self-reported health (1 = very poor, 5 = very good)	3.97	(0.71)	3.71	(0.74)	≤ 0.01

The values shown in the table are either means with standard deviations or numbers (*n*) with percentages (%) relating to the total sample or mean values with standard deviations.

## Measures

### Demographic characteristics and self-reported health

We measured previously identified demographic characteristics associated with participation or willingness to participate in a biobank, such as marital status [6, 12], education level [8–11], religion [10, 13], residential areas [12] and self-reported general health [8, 14]. Work status was included as additional indicator of socioeconomic status.

### Prosocial intrapersonal characteristics

#### Prosocial orientation and values

We applied two measures to determine respondents’ prosocial orientations. The first was their actual prosocial behaviour reflected in their organ and blood donor status and the frequency of their charitable donations. The second was the degree to which respondents cared about others’ outcomes in resource allocation choices, which was measured using six key items of the Social Value Orientation (SVO) scale. This scale is designed to measure the magnitude of individuals’ general concern for others [33]. Replicating previous research, our results indicated excellent reliability with *α* > 0.90 [34].

We used eleven items based on the theory of basic values to assess motivations of self-interest and beyond [35]. Moreover, we applied an adapted version of the Environmental Personal Values Questionnaire (E-PVQ) scale [26]. Although our focus primarily was on behaviour in the context of healthcare, we investigated values related to a wider context, e.g., about the environment. This might distinguish individuals acting based on their general concern for others, themselves and a common environment. In addition to the original subscales measuring hedonic values (individual pleasure, e.g., “it is important to have fun”) and biospheric values (concern about the environment, e.g., “it is important to protect the environment”), we included a subscale on healthspheric values (health and healthcare concerns, e.g. “it is important to live a fit life”). The reliability was good with *α* > 0.80 on all subscales, but they had a strong intercorrelation (Table 2).

**Table 2:** Correlation table.

Variable	1	2	3	4	5	6	7	8	9	10	11	12
1. Age	1											
2. Educational level (1 = low, 2 = moderate, 3 = high)	**−0.21**	1										
3. Self-reported health	**−0.07**	**0.15**	1									
4. Frequency of charitable donations	**0.16**	**0.06**	**0.05**^**S**^	1								
5. Social Value Orientation score	0.01	**0.06**	0.02	**0.15**	1							
6. Healthsperic values	**0.17**	−0.02	**0.26**	**0.14**	**0.12**	1						
7. Hedonic values	**−0.07**	**−0.05**	**0.16**	**0.07**	**0.09**	**0.71**	1					
8. Biospheric values	**0.23**	−0.01	**0.12**	**0.18**	**0.14**	**0.73**	**0.54**	1				
9. Societal trust	0.02	**0.22**	**0.22**	**0.18**	**0.13**	**0.20**	**0.13**	**0.17**	1			
10. TDM&H/government	**−0.04**^**S**^	**0.11**	**0.12**	**0.11**	**0.12**	**0.19**	**0.19**	**0.16**	**0.42**	1		
11. TDM&H/commercial enterprises	**0.06**	**−0.14**	0.03	**0.07**	**0.05**	**0.05**	0.01	0.02	**0.24**	**0.34**	1	
12. TDM&H/commercial researchers	**0.04**^**S**^	**0.05**	**0.07**	**0.09**	**0.08**	0.02	−0.01	**0.04**^**S**^	**0.34**	**0.47**	**0.40**	1

Bold is significant with *p* < 0.01; bold^S^ is significant with *p* < 0.05.

#### Societal trust

Societal trust was measured based on the framework proposed by Mayer et al. [36]. We used six items reflecting trust in the government and trust in other citizens on a five-point Likert-scale, e.g., “the government can take care of her citizens”; “the government acts with good intentions”; “people are trustworthy”. The six items tap into various aspects of societal trust, namely, competence, good intentions, and trustworthiness [36]. The combined scale had a good reliability (*α* = 0.86).

In addition, five items measured trust in a domain-specific way (i.e., referring to large-scale data repository), namely, trust in different types of research organizations their data management and handling (i.e., hospital, government, university, large-sized commercial enterprises, and medium-sized enterprises (SMEs)). For example, we used items “I believe that the hospital correctly, adequately and fairly store and treat my personal data”. Besides trust in organizations as entity, we measured trust in specific common research employees their data management and handling with four items (i.e., hospital researcher, university researcher, market researcher, polling researcher). For example, an item was “I trust employees of the hospital conducting research with a correct, fair and careful approach”. We conducted a principal component analysis with an oblimin rotation, due to several high intercorrelations (*r* > 0.50). The analysis showed that the items could be reduced to three factors explaining 71.90% of the total variance. The first factor, with four items, was labelled “Trust in data management and handling of government” (including researchers’ data practices within its institutes) and explained 45.74% of the total variance (*α* = 0.83). The second factor, “Trust in data management and handling of commercial organizations”, comprised two items and explained 14.24% variance (*α* = 0.65). The third factor, “Trust in data management and handling of commercial researchers”, comprised two items and explained 11.92% of the total variance (*α* = 0.47). Table 2 shows that the correlations between components were low to moderate (*r* < 0.40).

### Data analysis

We calculated descriptive statistics for demographic and psychological variables, applying the chi-square test or independent *t*-tests to calculate differences in means between participants and refusers where appropriate. We performed multivariate binary logistic regression analyses with a manual entering strategy to build respondents’ profiles based on their psychological characteristics while controlling for demographic characteristics. All analyses were conducted using SPSS, Version 25.0 [37].

## Results

### Demographic characteristics and self-reported health

Table 1 shows that more participants than refusers had registered partnerships including marriages (79.5 vs. 66.6%, *p* < 0.01). There was a positive though non-significant trend for having a job (55.8 vs. 50.7%, *p* = 0.07). While participants reported better general health status (3.97 vs. 3.71, *p* < 0.01), refusers had a higher educational level (46.8 vs. 41.4%, *p* < 0.01), were more commonly urban dwelling (77.2 vs. 73%, *p* < 0.01) and less religious (37.2 vs. 34.7%, *p* < 0.01).

### Prosocial intrapersonal characteristics

#### Prosocial orientation and values

Table 3 shows that compared to refusers, participants were more commonly contributors to charities (2.68 vs. 2.44, *p* < 0.01), blood donors (11.7 vs. 5.4%, *p* < 0.01) and organ donors (55.8 vs. 36.7%, *p* < 0.01). This finding reflects the stronger concerns about others among participants, as indicated by their SVO scores compared with refusers’ scores (32.25 vs. 29.95, *p* < 0.01). Moreover, they appeared to be more concerned about health, a healthy lifestyle (3.99 vs. 3.75, *p* < 0.01), and their own pleasure (4.25 vs. 4.09, *p* < 0.01) than about their environment (3.76 vs. 3.83, *p* < 0.01).

**Table 3:** Prosocial intrapersonal characteristics of participants and refusers of the Lifelines biobank.

	Participants		Refusers		*p*
Frequency of donations to charities	2.68	(1.08)	2.44	(1.04)	≤0.01
Blood donor	306	(11.7%)	22	(5.4%)	≤0.01
Organ donor	1460	(55.8%)	149	(36.7%)	≤0.01
Social Value Orientation score	32.25	(14.19)	29.94	(13.85)	≤0.01
Values					
Healthspheric	3.99	(0.66)	3.73	(0.73)	≤0.01
Hedonic	4.25	(0.65)	4.09	(0.70)	≤0.01
Biospheric	3.76	(0.75)	3.83	(0.75)	≤0.01
Societal trust	3.25	(0.63)	3.02	(0.74)	≤0.01
TDM&H/government	3.82	(0.54)	3.62	(0.71)	≤0.01
TDM&H/commercial enterprises	2.73	(0.73)	2.74	(0.76)	=0.81
TDM&H/commercial researchers	3.34	(0.72)	3.44	(0.67)	≤0.01

The values in the table are either means with standard deviations or numbers (*n*) with percentages (%) relating to the total sample.

*TDM&H* trust in data management and (data) handling.

#### Societal trust

Participants had higher levels of trust in the government and in other citizens compared to refusers (3.25 vs. 3.14, *p* < 0.01), especially regarding the government’s management and handling of data including researchers’ data management practices within its institutes (3.82 vs. 3.62, *p* < 0.01). However, participants were less trusting of researchers’ data handling and management practices within commercial enterprises (3.34 vs. 3.44, *p* < 0.01). There was no difference in levels of trust concerning commercial enterprises among participants and refusers (2.73 vs. 2.74, *p* > 0.50).

### Main analysis

We started our main analysis by testing the assumptions of our logistic regression, which were all met. First, there were no indications of multicollinearity; all VIF scores were <10.0 [38]. Second, some of the continuous variables showed a non-linear relationship of the independent variable to their log odds, as shown by the Box Tidwell test [39]. These variables were adjusted for their non-linear relationship as long as the model fit significantly improved (Table 4). Lastly, the sample size was considered sufficient applying the rule of thumb suggested by Peduz and collaborators of *n* > 10 × 14/0.1 = 1400 and not finding expected cell counts < 5 [40].

**Table 4:** Results of the logistic regression of participants and refusers of the Lifelines biobank.

Variable	Step	OR	95% CI OR	*p*	Pseudo R^2^ [R^2^ Cox and Snell; R^2^ Nagelkerke]
Age	1	1.01	[1.00, 1.02]	= 0.09	[0.03; 0.06]
Gender	1	0.90	[0.68, 1.19]	= 0.45	
Marital status	1	1.68	[1.24, 2.28]	< 0.01	
Educational level High vs. other	1	0.58	[0.42, 0.79]	< 0.01	
Educational level Low vs. other	1	1.77	[1.16, 2.71]	< 0.01	
Paid job (yes = 1, no = 0)	1	1.32	[094, 1.85]	= 0.11	
Religious (yes = 1, no = 0)	1	1.06	[0.79, 1.41]	= 0.72	
Residence (rural = 1, urban = 2)	1	0.81	[0.58, 1.12]	= 0.20	
Self-reported health	1	1.28	[1.04,1.57]	= 0.02	
Frequency of charitable donations	2	1.10	[0.95, 1.27]	= 0.20	[0.04; 0.07]
Blood donor	3	2.31	[1.35, 3.94]	< 0.01	[0.04; 0.08]
Organ donor	4	1.94	[1.45, 2.58]	< 0.01	[0.05; 0.10]
Social Value Orientation score	5	1.00	[1.00, 1.01]	= 0.37	[0.05; 0.10]
Healthsperic values	6a	–	–	= 0.02	[0.11; 0.20]
Healthsperic values^2^	6b			< 0.01	[0.13; 0.25]
Healthsperic values^3^	6c			< 0.01	[0.14; 0.27]
Hedonic values	7a	–	–	< 0.01	[0.15; 0.28]
Hedonic values^2^	7b			< 0.01	[0.17; 0.33]
Biospheric values	8a	–	–	< 0.01	[0.18; 0.35]
Biospheric values^2^	8b			< 0.01	[0.19; 0.36]
Societal trust	9	1.38	[1.08, 1.77]	< 0.01	[0.19; 0.37]
TDM&H/government	10a	–	–	< 0.01	[0.20; 0.38]
TDM&H/government^2^	10b			< 0.01	[0.20; 0.38]
TDM&H/commercial enterprises	11	0.75	[0.61, 0.93]	< 0.01	[0.20; 0.38]
TDM&H/commercial researchers	12	0.57	[0.45, 0.73]	< 0.01	[0.21; 0.40]

Test statistics multivariate model final step: *χ*^2^(26) = 615.414, *p* < 0.01.

*OR* odds ratio (not reported if transformated), *CI* confidence interval.

The results of the multivariate logistic regression analyses of potential predictors of participation revealed that our model performed well with a significant goodness of fit (*χ*^2^(26) = 615.414, *p* < 0.01). The fit of our model was good with Pseudo R^2^ being 0.40 (Table 4). Differences between participants and refusers were primarily explained by prosocial intrapersonal characteristics, while demographic characteristics provided only limited explanation. Notably, in our model, having a (registered) partner and a low educational level were relevant but slightly weaker predictors among the demographic variables. Low educational level significantly increased the odds to be participant in Lifelines in comparison with moderate or high educational levels, while a high level compared to moderate and low level of education had a smaller yet significant effect. In contrast, residence did not any longer contribute to differences between participants and refusers, once other predictors were taken into account (OR = 0.76, *p* = 0.10). A higher self-reported health significantly predicted participation in our model (OR = 1.28, *p* = 0.02).

Healthspheric values were the strongest predictors of participation in the simultaneous analyses of demographic and psychological predictors. This finding is reflected in the significant predictors of blood donor status (OR = 2.31, *p* < 0.01) and organ donor status (1.94, *p* < 0.01). The lower scores in biospheric values of participants compared to refusers were robust in our multivariate analyses (3.76 vs. 3.83, *p* < 0.01). Table 4 shows that associations of SVO score (OR = 1.00, *p* = 0.37) and frequency of charitable donations (OR = 1.10, *p* = 0.20) with participation in the univariate analyses were not robust in the multivariate model. Although societal trust as well as trust in data management and handling by the government independently contributed to the prediction of participation, the level of trust in data management and handling by commercial researchers was a key predictor of participation (OR = 0.57, *p* = 0.01). There is a clear lack of trust among participants, while refusers seem to have less issues in trusting commercial researchers and personal data.

## Discussion

In this study, we examined demographic and psychological predictors of (non-)participation within a comparative analysis of biobank’s participants and refusers. The results indicated that prosocial intrapersonal variables were stronger predictors of participation in biobanking than demographic variables. Specifically, health-related values and trust in management and handling of data by researchers appeared as key factors in distinguishing participants from refusers.

Our results add to previous findings on the importance of prosocial values and societal trust in participation in biobanking [3, 4, 6, 13, 18, 41]. The appraisal of the context of biobanking, that is reflected in prosocial values and trust in society or government, was a key factor for participation. First, we found opposite associations of healthspheric and biospheric values with participation. This indicates that refusers prioritize health or healthcare differently. The differences in donor status for blood or organs support this explanation, since the odds ratio to be blood or organ donor was smaller for refusers. Even though studies showed that motives for research participation are prosocial [18, 42], we did not find a stronger general “prosociality” score of participants compared to refusers when taking into account all other factors. Similarly, a recent psychological study with college majors found associations of organ donation with a medicine major, but only positive trends for prosocial SVO score [43].

Second, the opposite associations of trust in data management and handling of government and of commercial researchers with participation suggest that trust could simultaneously work as facilitator and barrier for participation in biobanking depending on the context. Refusers in our study had less societal trust yet more trust in commercial researchers compared to participants. This lower variance in refusers’ trust in public vs. private context might indicate a more sceptic yet homogenic perception regardless of context. Two other studies found similar variances of trust in these contexts among non-participants [5, 30]. Moreover, our results extend findings of a recent international comparison of trust and intention to participate in genomic research and big data initiatives, since we show how participants and refusers perceive systems of authority differently, especially in research [5, 6]. Our findings argue for a context-specific understanding of trust [30, 44]. Similar to previous findings, trust in biobanks depended on its context, e.g., biobanks’ funding stream or accessibility for third-party researchers [3, 4, 6, 45].

In our analyses, only educational level and being with partner were relevant demographic predictors of participation when taking into account prosocial intrapersonal characteristics. This supports previous findings on associations of participation with higher educational level and having a marital status [4–6]. First, a recent multinational study about genetic data sharing showed a similar effect for marital status on participation intention [6]. Recruitment aiming to include hereditability might be more prone for inclusion of citizens with partner. Recruitment strategies need to address this bias separately, given its robust effect in our multivariate model.

Second, we found that a higher education was associated with a higher probability of refusing to participate, regardless of levels of trust. This finding contradicts the literature showing that higher educational level is associated with participation [5, 6], yet confirms a recent study showing a highest willingness to participate in biobank research for middle educational level [22]. Possibly, our finding can be explained as a methodological issue, since the refusers sample underrepresented low educational level, while the participants sample represented this group more accurately. It is, however, also possible that psychological or social mechanisms explain this finding; samples with a mandatory sampling strategy provide better representativeness than voluntary samples [46]. The peer/family pressure for three generations of one family participating in Lifelines might have given the Lifelines’ sample a mandatory character, which was not the case for the refusers panel. Another explanation might be a variance in motives for (non-)participation among higher and lower educated people, or a relevant variance in their social networks, including other people participating. Future research should investigate how educational level and marital status independently might affect participation and refusal of participation via these or other psychological or social factors.

### Implications

Our findings suggest that biobanks seeking to recruit participants should consider multiple prosocial intrapersonal characteristics within their recruitment strategies. Our findings indicate that healthspheric, biospheric values and trust in government are strongly associated with participation in large-scale biomedical research. Therefore, emphasizing contextual prosocial benefits of participation and increasing the trustworthiness of biobanks as being independent from the government may facilitate the recruitment of new participants.

To retain participants, it may be wise to be cautious regarding collaborations with commercial enterprises because of participants’ distrust of commercial researchers. The lack of trust in commercial enterprises is a general concern for recruitment and retention of participants. Our results highlight the importance of establishing governance and accountability structures that enhance trust in the biobanking context [45]. Solutions might be found in introducing trustworthy researchers as gatekeepers to data, in particular hospital researchers [6]. Although the role of societal trust has been acknowledged, research practices that strengthen trust in different private and public contexts merit greater attention. This consideration is particularly pressing in the field of personalized medicine, in which the collaborative management and handling of data, entailing diverse contexts, have sparked serious concerns over data use [18, 29].

### Limitations

Although our study has provided insights into differences between participants and refusers of medical research, it has some limitations. Sampling bias may have occurred, as individuals who refused to participate in the biobank were nevertheless willing to complete an online questionnaire on this topic. Consequently, they may not be representative of all individuals unwilling to participate in biobanks or data repositories. Our sample of refusers was experienced and aware of commercial research or the procedures for research in general, which may explain their higher levels of trust in commercial researchers [4]. Nevertheless, our findings provide valuable inputs for recruitment strategies and effective methods for data repository.

## Conclusion

In conclusion, we have shown that participants and refusers of biobanking can be distinguished according to their prosocial intrapersonal characteristics. Our findings may contribute to improving recruitment strategies by incorporating relevant values and/or highlighting prosocial benefits. Moreover, they underline the need to address trust issues in collaborations between data repositories and commercial companies. Future research should explore how prosocial intrapersonal characteristics drive participation and withdrawal decisions.

## Supplementary information

Supplementary Table 1
