# Supplementary material for: Demographic and prosocial intrapersonal characteristics of biobank participants and refusers: the findings of a survey in the Netherlands
Source: Eur J Hum Genet. 2020 Jul 31;29(1):11–9. doi: 10.1038/s41431-020-0701-1 (PMC7852517; doi:10.1038/s41431-020-0701-1)
Supplement: Supplementary file 1 — Supplementary Table 1 [file 41431_2020_701_MOESM1_ESM.docx]

Supplementary Table 1 Demographic characteristics and self-reported health of (non)participants of a Dutch population-based biobank

|  | Participants | | Refusers | | Other nonparticipants | | |
| --- | --- | --- | --- | --- | --- | --- | --- |
| Age | 56.27 | (15.88) | 56.09 | (14.18) | | 56.73 | (15.78) |
| Gender |  |  |  |  | |  |  |
| Male | 1291 | (50.5%) | 179 | (48.5%) | | 654 | (51.8%) |
| (Female | 1264 | (49.5%) | 190 | (51.5%) | | 609 | (48.2%) |
| Marital status |  |  |  |  | |  |  |
| Registered partner | 2,078 | (79.5%) | 269 | (66.6%) | | 937 | (70.8%) |
| No registered partner | 536 | (20.5%) | 134 | (33.4%) | | 386 | (29.2%) |
| Educational level |  |  |  |  | |  |  |
| High | 1,083 | (41.4%) | 189 | (46.8%) | | 711 | (54.1%) |
| Moderate | 938 | (35.9%) | 50 | (37.1%) | | 477 | (36.3%) |
| Low | 559 | (21.7%) | 60 | (14.9%) | | 127 | (9.7%) |
| Paid Job | 1,460 | (55.8%) | 205 | (50.7%) | | 644 | (48.7%) |
| Religious | 972 | (37.2%) | 140 | (34.7%) | | 504 | (39.1%) |
| Residence |  |  |  |  | |  |  |
| Rural | 2,019 | (77.2%) | 295 | (73.0%) | | 982 | (75.2%) |
| Urban | 537 | (20.5%) | 102 | (25.2%) | | 324 | (24.8%) |
| Self-reported health  (1= very poor, 5 = very good) | 3.97 | (0.71) | 3.71 | (0.74) | | 3.71 | (0.73) |

*Note:* The values shown in the table are either means with standard deviations or numbers (n) with percentages (%) relating to the total sample or mean values with standard deviations*.*
